# Identification of UAP1L1 as tumor promotor in gastric cancer through regulation of CDK6

**DOI:** 10.18632/aging.103050

**Published:** 2020-04-20

**Authors:** Jing Qi, Sheng Liu, Weihang Liu, Gaoqiang Cai, Guoqing Liao

**Affiliations:** 1 Department of Gastrointestinal Surgery, Xiangya Hospital Central South University, Changsha 410008, Hunan, China

**Keywords:** UAP1L1, CDK6, gastric cancer, cell proliferation, cell apoptosis

## Abstract

Gastric cancer (GC) is one of the most commonly diagnosed malignancies in digestive tract and its underlying molecular mechanism is still not clear, so we aimed to reveal the relationship between GC and UDP-GlcNAc pyrophosphorylase-1 like 1 (UAP1L1). The detection of UAP1L1 expression in GC tumor and normal tissues was accomplished by immunohistochemistry and demonstrated the upregulation of UAP1L1 in GC, which was statistically associated with tumor grade. GC cell models constructed *via* transfection of UAP1L1-silencing/overexpressing lentiviruses were employed for evaluating the effects of UAP1L1 knockdown/overexpression on GC *in vitro* and *in vivo*. The results indicated that UAP1L1 played important role in development of GC through regulating cell proliferation, colony formation, cell apoptosis and cell migration. Subsequently, CDK6 was identified as a potential target in UAP1L1 induced regulation of GC, downregulation of which exhibited similar inhibition effects on GC with UAP1L1. Moreover, it was demonstrated that the promotion of GC by UAP1L1 overexpression could be significantly attenuated or even reversed by simultaneously silencing CDK6. In conclusion, UAP1L1 was reported to be a tumor promotor in the development and progression of GC which may exert its role through regulating CDK6 and may act as a candidate of therapeutic target in treatment.

## INTRODUCTION

Gastric cancer is one of the most common malignant tumors and severely endangers human health [1]. According to the statistical data, gastric cancer possesses the fifth highest morbidity among all malignant tumors, and occupies the third largest proportion of cancer-related death which is only lower than lung cancer and colorectal cancer [2, 3]. Up to now, surgical resection is still recognized as the only radical treatment for gastric cancer [4, 5]. However, due to the difficulty of early diagnosis and the trend of reoccurrence and metastasis after surgical treatment, the 5-year survival rate of gastric cancer is still less than 30% worldwide [6, 7]. Recently, with the deepening of the research on the molecular mechanism of gastric cancer, which is a complex involving multiple genes and factors, molecular targeted therapy has been increasingly used in the treatment of gastric cancer [8–11]. Therefore, the exploration of novel target molecules, which express abnormally in gastric cancer, is of great significance in the development of more effective targeted therapy for gastric cancer treatment and would benefit gastric cancer patients.

Uridine-diphospho-*N*-acetylglucosamine (UDP-GlcNAc) is an essential precursor of *N*- or *O*-linked glycosylation in important metabolic processes [12]. It has been identified as a substrate of chitin synthase, the product of which is essential for fungal cell wall [13], and it also plays important roles in the biosynthesis of cell wall peptidoglycan and the disaccharide moiety of lipid A in bacteria [14–16]. *O*-GlcNAc transferase (OGT) is a single enzyme which is responsible for catalyzing UDP-GlcNAc to serines and threonines of various protein substrates [17]. UAP1 (UDP-GlcNAc pyrophosphorylase-1) is an essential enzyme in the biosynthesis of UDP-GlcNAc through regulating the process from UTP and GlcNAc1P to pyrophosphate and UDP-GlcNAc or its reverse form [18, 19]. UAP1L1 (UAP1 like 1) is a protein with 59% sequence similarity as UAP1 [20]. Considering the important role played by glycosylation in various biological processes and the involvement of changes in protein glycosylation in malignant tumors, it was predicted that UAP1L1 would also participate in the development and progression of human cancers [21]. However, research concerning the functions of UAP1L1 in cancer is still rarely observed and its association with gastric cancer is still unclear.

In this study, we detected the differential expression of UAP1L1 between gastric cancer and normal tissues, revealing the upregulated UAP1L1 level in gastric cancer and the positive relationship between high UAP1L1 expression and more advanced tumor grade. It was further demonstrated *in vitro* and *in vivo* that changes in the expression of UAP1L1 has significant regulatory effects on gastric cancer cell proliferation, apoptosis and motility, and could disturb the tumorigenicity of gastric cancer cells as well as slow down the tumor growth. Moreover, this study also aimed to explore the mechanism, by which UAP1L1 promotes the development and progression of gastric cancer, and identified CDK6 as a potential target of UAP1L1. Therefore, we provide powerful evidence of the involvement of UAP1L1 in gastric cancer, which may be used as a novel therapeutic target in the treatment of gastric cancer.

## RESULTS

### UAP1L1 is upregulated in gastric cancer tissues and expressed in gastric cancer cells

**Figure 1:**
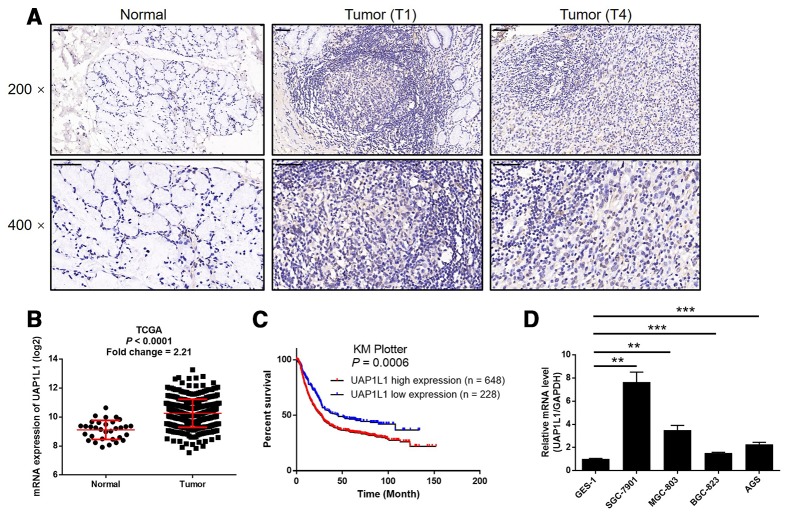
**UAP1L1 was upregulated in gastric cancer tissues and gastric cancer cells.** (**A**) The expression level of UAP1L1 was detected by IHC analysis in gastric cancer tissues and normal tissues (scale bar = 50 μm). (**B**) The mining of RNA-seq data of TCGA showed the upregulated mRNA expression of UAP1L1 in tumor tissues of gastric cancer patients compared with that in normal tissues. (**C**) The mining of prognosis data of KM plotter showed the significantly association between UAP1L1 high expression and shorter survival period of gastric cancer patients. (**D**) The mRNA expression of UAP1L1 in GES-1, BGC-823, SGC-7901, AGS and MGC-803 cell lines was detected by qPCR. The representative images were selected from at least 3 independent experiments. Data was shown as mean ± SD. *P < 0.05, **P < 0.01, ***P < 0.001.

In this study, we first investigate the expression of UAP1L1 in human gastric cancer tissues and compared with that in normal tissues to preliminarily estimate its role in gastric cancer. The results of IHC analysis showed that the expression level of UAP1L1 in tumor tissues was much higher than that in normal tissues, indicating that UAP1L1 may be involved in the development and progression of gastric cancer (Figure 1A and Table 1). Consistently, the RNA-seq data collected from TCGA-STAD database of The Cancer Genome Atlas (TCGA) also demonstrated a 2.21-fold higher UAP1L1 expression in tumor tissues compared with normal tissues (*P* < 0.001, Figure 1B). Further correlation analysis between UAP1L1 expression and tumor characteristics of gastric cancer patients showed its significant association with T stage (T infiltrate) (Table 2), which was also confirmed by Spearman rank correlation analysis (Supplementary Table 3). More importantly, the Kaplan-Meier survival analysis of the data collected from KM plotter database indicated that high expression of UAP1L1 was significantly associated with poorer prognosis of gastric cancer patients, as well as shorter survival period (*P* = 0.0006, Figure 1C). On the other hand, the expression of UAP1L1 in human gastric mucosal epithelial cell GES-1 and various types of gastric cancer cell lines was detected by qPCR. As shown in Figure 1D, despite of the differential expression level, the expression of UAP1L1 was found to be upregulated in gastric cancer cells compared with GES-1 cells. Altogether, these experimental results and bioinformatics revealed the involvement of UAP1L1 in the development and progression of gastric cancer.

**Table 1:** Expression patterns of UAP1L1 in gastric cancer tissues and normal tissues revealed in immunohistochemistry analysis.

**UAP1L1 expression**	**Tumor tissue**		**Normal tissue**	
	**Cases**	**Percentage**	**Cases**	**Percentage**
Low	13	32.5%	35	89.4%
High	27	67.5%	4	10.3%

*P* < 0.001.

**Table 2:** Relationship between UAP1L1 expression and tumor characteristics in patients with gastric cancer.

**Features**	**No. of patients**	**UAP1L1 expression**		***P* value**
		**low**	**high**	
All patients	40	13	27	
Age (years)				0.441
<35	18	7	11	
≥35	22	6	16	
Gender				0.892
Male	16	5	11	
Female	24	8	16	
Grade				0.393
1	1	1	0	
2	1	0	1	
3	37	12	25	
4	1	0	1	
T Infiltrate				0.006**
T1	12	7	5	
T2	3	2	1	
T4	25	4	21	
Lymphatic metastasis (N)				0.639
N0	15	4	11	
N1	6	2	4	
N2	7	3	4	
N3	12	4	8	
Stage				0.270
1	9	5	4	
2	11	3	8	
3	15	3	12	
4	5	2	3	
Tumor metastasis (M)				0.963
M0	34	11	23	
M1	6	2	4	

### UAP1L1 knockdown inhibited gastric cancer development *in vitro*

**Figure 2:**
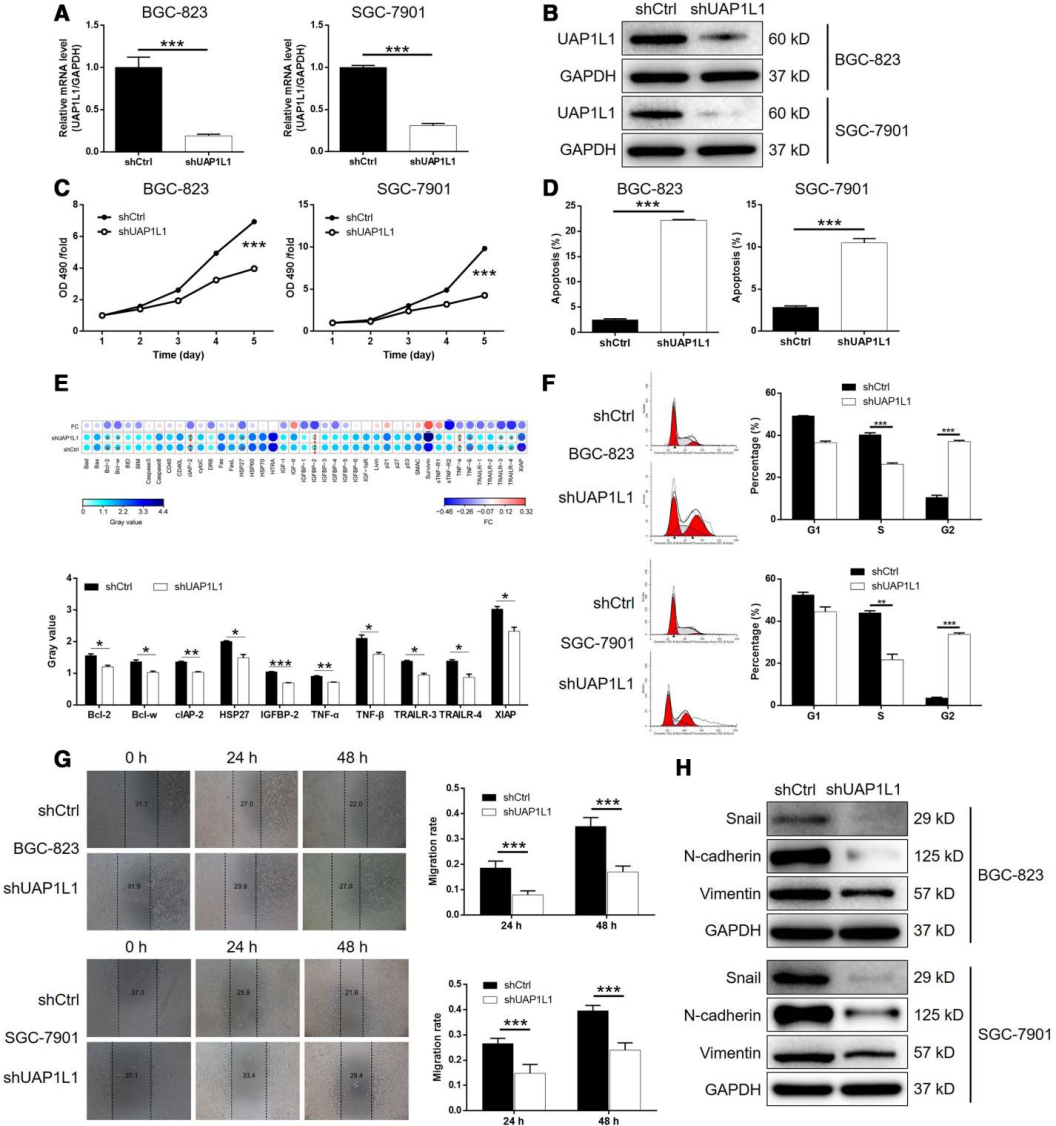
**UAP1L1 knockdown inhibited gastric cancer development *in vitro*.** (**A**, **B**) Cell models with or without UAP1L1 knockdown were constructed by transfecting shUAP1L1 or shCtrl. The knockdown efficiency of UAP1L1 in BGC-823 and SGC-7901 cells was assessed by qPCR (**A**) and western blotting (**B**). (**C**) MTT assay was employed to show the effects of UAP1L1 on cell proliferation of BGC-823 and SGC-7901 cells. (**D**) Flow cytometry was performed to detect cell apoptosis of BGC-823 and SGC-7901 cells with or without UAP1L1 knockdown. (**E**) Human Apoptosis Antibody Array was utilized to analyze the regulatory ability of UAP1L1 on expression of apoptosis-related proteins in SGC-7901 cells. (**F**) Cell cycle distribution was estimated in BGC-823 and SGC-7901 cells with or without UAP1L1 knockdown. (**G**) The effects of UAP1L1 on cell migration ability of BGC-823 and SGC-7901 cells were evaluated by wound-healing assay. (**H**) The expression of EMT-related proteins including Snail, N-cadherin and Vimentin was detected by western blotting in BGC-823 and SGC-7901 cells of shUAP1L1 and shCtrl groups. The representative images were selected from at least 3 independent experiments. Data was shown as mean ± SD. *P < 0.05, **P < 0.01, ***P < 0.001.

In order to explore the role played by UAP1L1 in the development and progression of gastric cancer, lentiviruses expressing shRNA targeting UAP1L1 (shUAP1L1) or shCtrl (as negative control) were prepared and used to knockdown the expression of UAP1L1 in BGC-823 and SGC-7901 cells with a transfection efficiency of >80% (Supplementary Figure 1). As shown in Figure 2A, the mRNA level of UAP1L1 was decreased by 81.0% (*P* < 0.001) and 69.2% (*P* < 0.001) in BGC-823 and SGC-7901 cells, respectively. The depletion of UAP1L1 was also verified by detecting its protein level in BGC-823 and SGC-7901 cells by western blotting (Figure 2B). Next, it was demonstrated that gastric cancer cells with downregulated expression of UAP1L1 (shUAP1L1) exhibited significantly slower proliferation rate than the shCtrl group (*P* < 0.001, Figure 2C). In consistent, the apoptotic cell percentage in BGC-823 and SGC-7901 cells with UAP1L1 knockdown was 8.9-fold and 3.7-fold higher than the cell transfected with shCtrl (*P* < 0.001, Figure 2D). Subsequently, a Human Apoptosis Antibody Array was performed on SGC-7901 cells with or without UAP1L1 to manifest the regulatory effects of UAP1L1 knockdown on apoptosis-related proteins, which demonstrated the downregulation of Bcl-2, Bcl-w, clAP-2, HSP27, IGFBP-2, TNF-α, TNF-β, TRAILR-3, TRAILR-4 and XIAP (*P* < 0.05, Supplementary Figure 2A and 2E). Moreover, the detection of cell cycle distribution clarified that knockdown of UAP1L1 induced the arrest of cell cycle in G2 phase and decreased the percentage of cells in S phase (*P* < 0.01, Figure 2F), by which may UAP1L1 promotes cell proliferation and cell apoptosis. Otherwise, the results of wound-healing assay showed the significantly suppressed cell migration ability of BGC-823 and SGC-7901 cells in shUAP1L1 group (*P* < 0.001, Figure 2G), which was further rationalized by the downregulated expression of EMT-related proteins including Snail, N-cadherin and Vimentin, as well as upregulation of E-cadherin and ZO-1 (Figure 2H and Supplementary Figure 2B). Collectively, the *in vitro* studies clearly validated that UAP1L1 plays an important role in the development and progression of gastric cancer.

### The potential of CDK6 as the downstream of UAP1L1 in the regulation of gastric cancer

**Figure 3:**
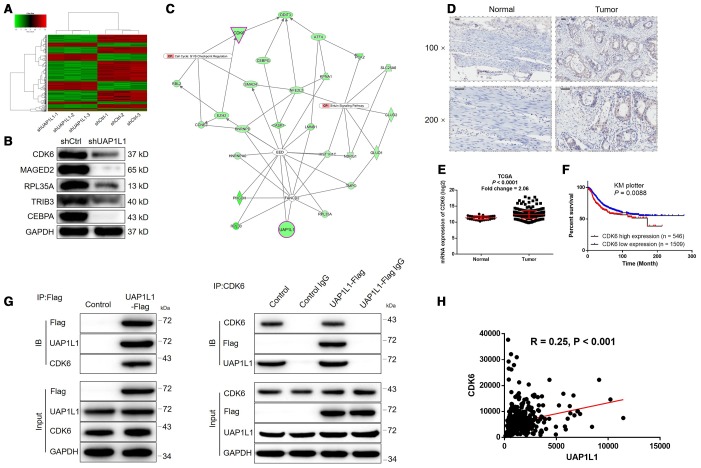
**The exploration and verification of downstream underlying UAP1L1 induced regulation of gastric cancer.** (**A**) A PrimeView Human Gene Expression Array was performed to identify the differentially expressed genes (DEGs) between shUAP1L1 and shCtrl groups of SGC-7901 cells. (**B**) Western blotting was used to detect the expression of several selected DEGs in SGC-7901 cells with or without UAP1L1. (**C**) A UAP1L1 associated interaction network constructed by IPA analysis revealed the potential linkage between UAP1L1 and CDK6. (**D**) The expression of CDK6 in gastric cancer tissues and normal tissues was evaluated by IHC analysis (scale bar = 50 μm). (**E**) Data mining of expression profiling in TCGA database showed the upregulated expression of CDK6 in gastric cancer tissues compared with normal tissues. (**F**) Data mining of prognosis in KM plotter database showed that patients with higher expression of CDK6 suffered from shorter survival period. (**G**) The direct interaction between UAP1L1 and CDK6 was demonstrated by co-immunoprecipitation. (**H**) Data mining of expression profiling in TCGA database revealed the positive correlation between UAP1L1 and CDK6 in gastric cancer tissues. The representative images were selected from at least 3 independent experiments. Data was shown as mean ± SD. *P < 0.05, **P < 0.01, ***P < 0.001.

Given the clear-cut role of UAP1L1 in the development of gastric cancer, we next explored the downstream mechanism of its regulatory effects on gastric cancer. A PrimeView Human Gene Expression Array was performed to identify the differentially expressed genes (DEGs) between shUAP1L1 and shCtrl groups of SGC-7901 cells. Totally 610 DEGs were identified based on the threshold of absolute fold change > 2 and *FDR* < 0.05, among which 200 DEGs were upregulated, and the other 410 DEGs were downregulated in shUAP1L1 group (Figure 3A, Supplementary Figure 3A, 3B). Subsequently, all the DEGs were enriched in canonical signaling pathway and IPA disease and function by IPA analysis (Supplementary Figure 3C, 3D, Supplementary Figure 4 showed the significantly enriched glioma signaling which includes CDK6). Combining all the above results, 20 DEGs were selected for qPCR detection in SGC-7901 cells (Supplementary Figure 3E) and 5 of them were subjected to western blotting for further verification (Figure 3B), indicating the downregulation of CDK6 in UAP1L1 knockdown cells. Subsequently, high-content screening in which cells were infected with lentivirus expressing corresponding shRNAs showed that knockdown of CDK6 significantly inhibited SGC-7901 cell proliferation (Supplementary Figure 5A, 5B). The IPA analysis of UAP1L1 associated interaction network also illustrate the potential linkage of UAP1L1 and CDK6 (Figure 3C). IHC analysis (Figure 3D) and TCGA data mining (*P* < 0.0001, Figure 3E) were simultaneously performed to detect CDK6 expression in gastric cancer tissues and compared with that in normal tissues, both showing the upregulated expression of CDK6 in gastric cancer. As shown in Figure 3F, the prognosis information collected from 2055 gastric cancer patients in KM plotter database constructed the relationship between CDK6 high expression and relatively short survival period (*P* = 0.0088). More importantly, the direct UAP1L1-CDK6 interaction and positive correlation between them were confirmed by co-IP (Figure 3G) and TCGA data mining (Figure 3H), respectively. Finally, similar with UAP1L1, the expression of CDK6 was found to be significantly higher in gastric cancer cells compared to GES-1 cells (Supplementary Figure 5C).

### Knockdown of CDK6 blocked development of gastric cancer *in vitro*

**Figure 4:**
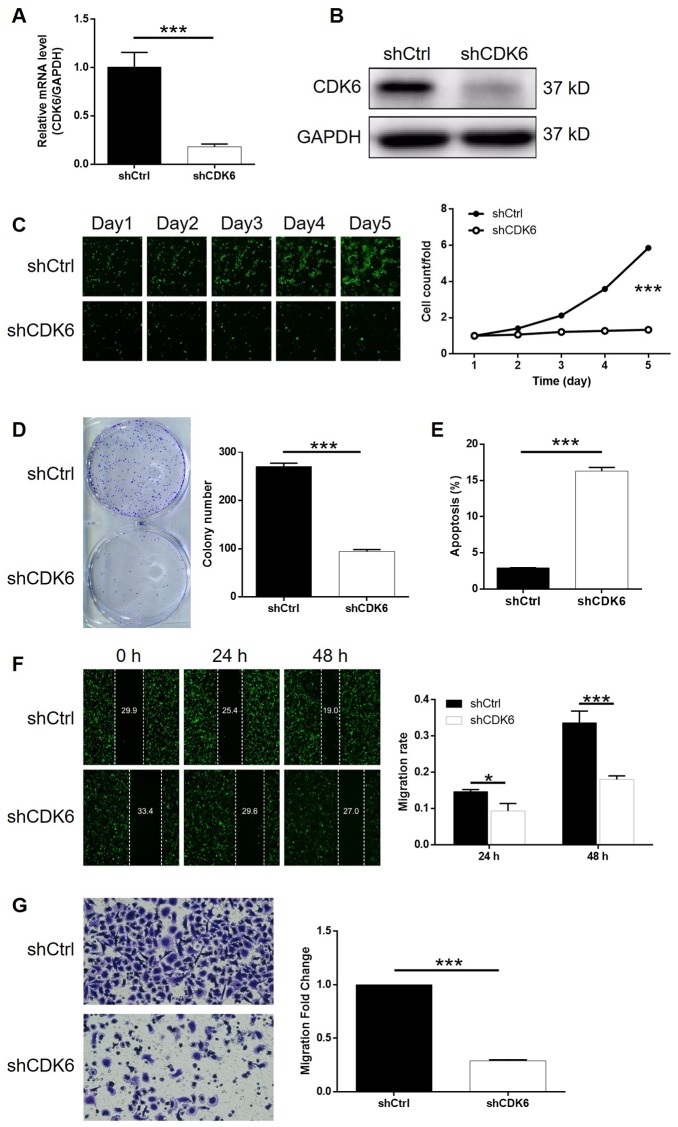
**CDK6 knockdown inhibited gastric cancer development *in vitro*.** (**A**, **B**) Cell models with or without CDK6 knockdown were constructed by transfecting shCDK6 or shCtrl. The knockdown efficiency of UAP1L1 in SGC-7901 cells was assessed by qPCR (**A**) and western blotting (**B**). (**C**) Celigo cell counting assay was employed to show the effects of CDK6 on cell proliferation of SGC-7901 cells. (**D**) Colony formation assay was used to evaluate the ability of SGC-7901 cells with or without CDK6 knockdown to form colonies. (**E**) Flow cytometry was performed to detect cell apoptosis of SGC-7901 cells with or without CDK6 knockdown. (**F**, **G**) The effects of CDK6 on cell migration ability of SGC-7901 cells were evaluated by wound-healing assay (**F**) and Transwell assay (**G**). The representative images were selected from at least 3 independent experiments. Data was shown as mean ± SD. *P < 0.05, **P < 0.01, ***P < 0.001.

In order to make clear the role of CDK6 in the development of gastric cancer, cytofunctional experiments were conducted using similar methods as mentioned above following the construction of CDK6 knockdown SGC-7901 cells. The transfection and knockdown efficiencies were evaluated by a combination of fluorescence imaging, qPCR and western blotting (*P* < 0.001, Supplementary Figure 6A, 4A, 4B). The results of Celigo cell counting assay indicated that cell growth of SGC-7901 cell transfected with shCDK6 almost stagnated while that transfected with shCtrl grew normally (*P* < 0.001, Figure 4C). Moreover, number of colonies formed by the cells was counted after 14 days of culture, displaying a large difference between shCDK6 (less) and shCtrl (more) groups (*P* < 0.001, Figure 4D). The effects of CDK6 knockdown on cell apoptosis and cell cycle were also proved to be similar with UAP1L1 knockdown, which increased apoptotic cell percentage in shCDK6 group by approximate 6-fold (*P* < 0.001, Figure 4E) and significantly induced G2 phase arrest (*P* < 0.001, Supplementary Figure 6B). Finally, a combination of wound-healing assay and Transwell assay distinguished that SGC-7901 cells with CDK6 knockdown suffered from much weaker motility (*P* < 0.001, Figure 4F, 4G). In summary, CDK6 possessed similar regulatory effects on the development of gastric cancer with UAP1L1.

### CDK6 knockdown alleviated UAP1L1 overexpression induced regulation of gastric cancer

**Figure 5:**
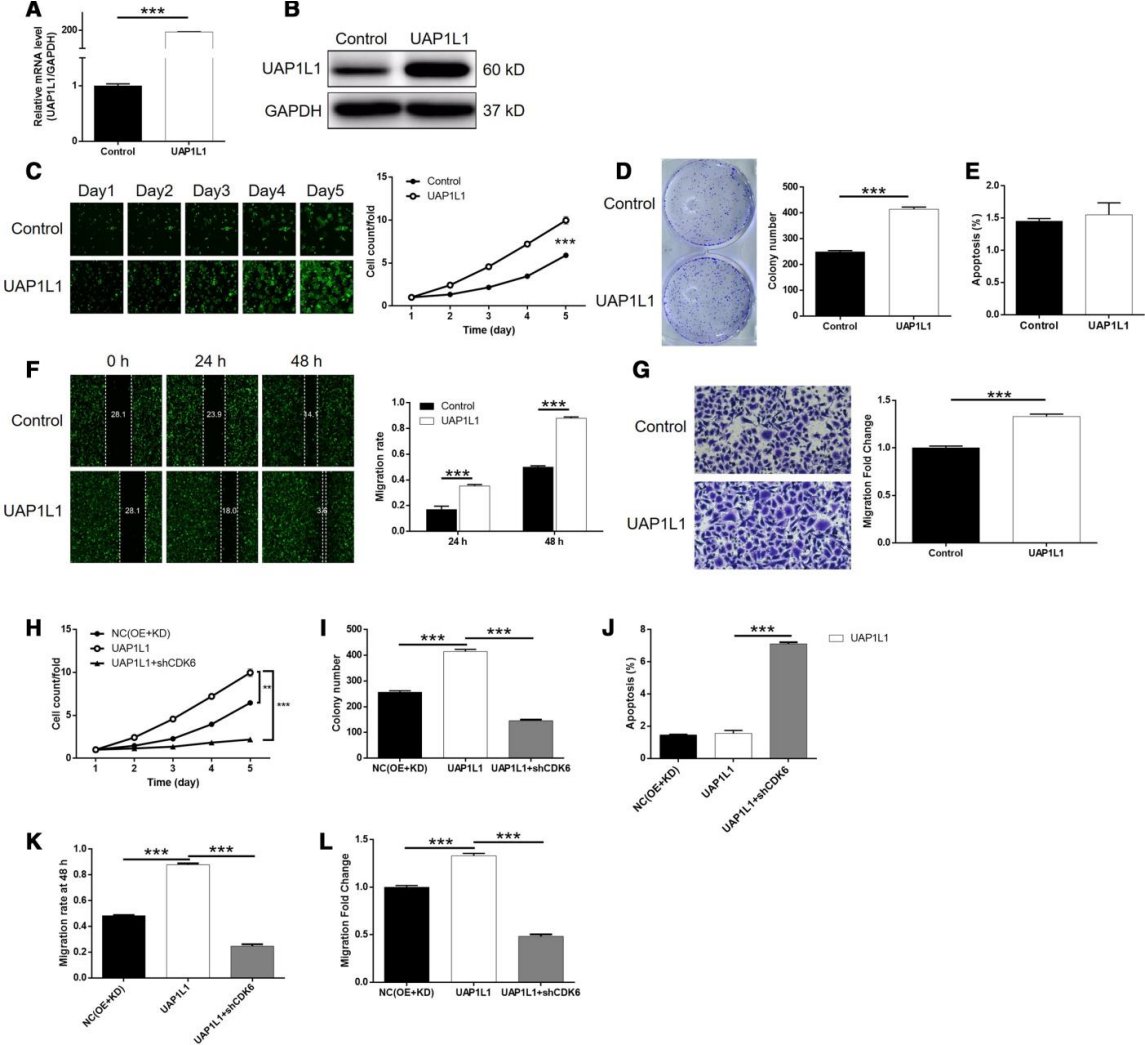
**Knockdown of CDK6 attenuated the effects of gastric cancer cells by UAP1L1 overexpression.** (**A**, **B**) Cell models with or without UAP1L1 overexpression were constructed by transfecting Control plasmids or UAP1L1 overexpression plasmids. The overexpression efficiency of UAP1L1 in SGC-7901 cells was assessed by qPCR (**A**) and western blotting (**B**). (**C**) Celigo cell counting assay was employed to show the effects of UAP1L1 on cell proliferation of SGC-7901 cells. (**D**) Colony formation assay was used to evaluate the ability of SGC-7901 cells with or without UAP1L1 overexpression to form colonies. (**E**) Flow cytometry was performed to detect cell apoptosis of SGC-7901 cells with or without UAP1L1 overexpression. (**F**, **G**) The effects of UAP1L1 on cell migration ability of SGC-7901 cells were evaluated by wound-healing assay (**F**) and Transwell assay (**G**). (**H**–**L**) SGC-7901 cells transfected with NC(OE+KD), UAP1L1 overexpression plasmids and simultaneous UAP1L1 overexpression plasmids and shCDK6 were subjected to the detection of cell proliferation by Celigo cell counting assay (**H**), colony formation (**I**), cell apoptosis by flow cytometry (**J**), cell migration by wound-healing assay (**K**) and cell migration by Transwell assay (**L**). The representative images were selected from at least 3 independent experiments. Data was shown as mean ± SD. *P < 0.05, **P < 0.01, ***P < 0.001.

We next constructed SGC-7901 cells with mere UAP1L1 overexpression and simultaneous UAP1L1 overexpression + CDK6 knockdown to investigate the synergistic effects of them on gastric cancer. First of all, the transfection of SGC-7901 cells by UAP1L1 overexpression plasmids (Supplementary Figure 7), as well as upregulation of UAP1L1 (*P* < 0.001, Figure 5A, 5B), significantly accelerate cell proliferation rate (*P* < 0.001, Figure 5C). In line with the results of cell proliferation assay, it was also found that UAP1L1 overexpression promoted the formation of colonies (*P* < 0.001, Figure 5D). Interestingly, we failed to observed the expected inhibition of cell apoptosis, which may be attributed to the low apoptosis rate in the shCtrl group (Figure 5E). Furthermore, UAP1L1 overexpression was shown to be capable of promoting cell motility of SGC-7901 cells based on the detection of wound-healing and Transwell assays (*P* < 0.001, Figure 5F, 5G). More importantly, the comparison of the results obtained from UAP1L1 group (UAP1L1 overexpression) and UAP1L1+shCDK6 group (UAP1L1 overexpression + CDK6 knockdown) group demonstrated that all the effects on cell proliferation, colony formation, cell apoptosis and cell migration by UAP1L1 overexpression could be alleviated or even reversed by CDK6 knockdown (*P* < 0.001, Supplementary Figures 7, 8, 5H–5L). In a word, these results clarified the promotion effects of UAP1L1 overexpression on gastric cancer, in which CDK6 may be involved.

### UAP1L1 knockdown inhibited tumor growth of gastric cancer *in vivo*

**Figure 6:**
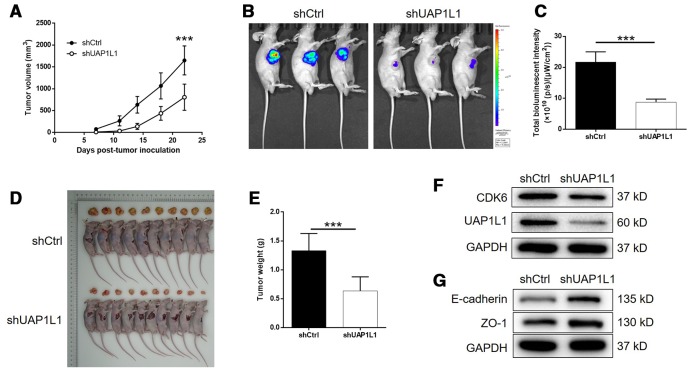
**UAP1L1 knockdown inhibited gastric cancer development *in vivo*.** (**A**) 7 days post injection of SGC-7901 cells with or without UAP1L1 knockdown, the volume of tumors formed in mice was measured and calculated at indicated time intervals. (**B**) *In vivo* imaging was performed to evaluate the tumor burden in mice of shUAP1L1 and shCtrl groups at day 22 post tumor-inoculation. (**C**) The bioluminescence intensity was scanned and used as a representation of tumor burden in mice of shUAP1L1 and shCtrl groups. (**D**, **E**) Mice were sacrificed at day 22 post injection, and the tumors were removed for collecting photos (**D**) and weighing (**E**). (**F**, **G**) The expression of UAP1L1, CDK6, E-cadherin and ZO-1 in xenografts was detected by western blotting. Data was shown as mean ± SD. *P < 0.05, **P < 0.01, ***P < 0.001.

For further verifying the influence of UAP1L1 knockdown on tumor growth *in vivo*, mice xenograft models were constructed through subcutaneous injection of SGC-7901 cells transfected with shUAP1L1 or shCtrl. The measurement of tumor volume started at day 7 post tumor-inoculation, the results of which illustrated the significantly slower growth rate of tumors formed in shUAP1L1 group (Figure 6A). The final volume (day 22) of tumors formed in shUAP1L1 group was calculated to be approximate 50% smaller than that in shCtrl group (*P* < 0.001, Figure 6A). On the other hand, the tumor burden of mice in shUAP1L1 and shCtrl groups was also assessed by *in vivo* imaging facilitated by injection of D-luciferin. The much stronger bioluminescence intensity in mice of shUAP1L1 groups than that of shCtrl group also proved the suppression of tumor growth by UAP1L1 knockdown *in vivo* (*P* < 0.001, Figure 6B, 6C). Moreover, the inhibited development of gastric cancer could also be affirmed by directly observing the removed tumors and the significantly lower weight of tumors removed from mice in shUAP1L1 group (*P* < 0.001, Figure 6D, 6E). Moreover, western blotting demonstrated the downregulated expression of UAP1L1 and CDK6, and upregulated expression of E-cadherin and ZO-1 in tumor tissues collected from shUAP1L1 group (Figure 6F, 6G). Therefore, all the above results strongly confirmed the role of UAP1L1 in the development and progression of gastric cancer *in vitro* and *in vivo*.

## DISCUSSION

Gastric cancer is one of the most commonly diagnosed malignancies in digestive tract with extremely high morbidity and mortality [3]. Although surgical resection is a well-acknowledged radical treatment for patients with early stage tumor, most gastric cancer patients are diagnosed with advanced tumor and could only be treated by a combination of chemotherapy and adjuvant therapies [7, 22, 23]. In the past decade, targeted therapy, which targets abnormally expressed factors in tumors, has attracted considerable attention because of its advantages including high efficacy, low side-effects. However, although several targeted drugs such as Trastuzumab [24] and Apatinib [25] have been used in the treatment of gastric cancer, the prognostic improvement of gastric cancer patients is still far from satisfactory. Therefore, the exploration of potential therapeutic target of gastric cancer may promote the development of molecular targeted therapy and has been a popular topic in the research field of gastric cancer [26, 27]. For example, a recent work published by Ma et al. [28] identified the important role of Eukaryotic translation initiation factor 3b (eIF3b) in proliferation and metastasis of gastric cancer, which also participated in the carcinogenic process of H. pylori. Another report indicated that RON/RONΔ160 could form a complex with β-catenin, thus promoting proliferation and migration of gastric cancer cells, which could be further enhanced by hypoxia as well as HIF-1α [29]. Otherwise, low expression of Dimethylarginine dimethylaminohydrolase 1 (DDAH1), which exhibited tumor suppression effects on gastric cancer, was also recognized as a potential predictor for more aggressive phenotypes and poor prognosis of gastric cancer [30].

UAP1 is an essential participant in *O*-linked GlcNAc modification in cytoplasm, nucleus and mitochondria, and also for *O*- or *N*-linked protein glycosylation in endoplasmic reticulum and the Golgi apparatus [31]. Given the important role of protein glycosylation in development of neoplastic disease, UAP1 was also found a critical regulator in prostate cancer. Through detecting UAP1 expression in 3261 prostate cancer tissue samples, Itkonen et al. found that UAP1 was highly overexpressed in prostate cancer and significantly associated with high concentration of UDP-GlcNAc. Moreover, their investigations further suggested that UAP1 could block the influence of *N*-linked glycosylation inhibitor on prostate cancer cells, which could be re-sensitized after silencing UAP1 [31]. UAP1L1 is another protein with 59% sequences identity to UAP1 [32]. The studies of Yang-Yen et al. indicated that, despite of the structural similarity between UAP1 and UAP1L1, they possess distinctly different functions in the synthesis of UDP-GlcNAc. Moreover, their studies also demonstrated the overexpression of UAP1L1 in HCC tissues compared with normal tissues and elucidated the promotion or inhibition of HCC development *in vitro* and *in vivo* by UAP1L1 overexpression or knockdown [32]. In spite of these, the role of UAP1L1 in human cancers, especially gastric cancer and the underlying mechanism are still not clear.

In this study, it was found that the expression of UAP1L1 in gastric cancer tissues was generally higher than that in normal tissues. Moreover, patients with relatively higher UAP1L1 expression tend to suffer from tumors in more advanced grade, indicating the potential role of UAP1L1 in the development of gastric cancer. Data mining of TCGA and KM plotter database not only verified the abnormal overexpression of UAP1L1 in gastric cancer, but also built a linkage between UAP1L1 high expression and poor prognosis. Subsequent *in vitro* experiments validated the role of UAP1L1 in gastric cancer via uncovering the inhibition of cell proliferation and cell motility, and the promotion of cell apoptosis induced by UAP1L1 knockdown. Furthermore, the regulation of cell apoptosis and cell migration was further understood by the alteration of apoptosis-related or EMT-related proteins caused by UAP1L1 knockdown. The observation and measurement of tumor-bearing mice models further make clear the promotor role of UAP1L1 in the development and progression of gastric cancer, with an unclear mechanism.

It is well-known that the dysfunction of cell cycle is an important inducement of tumorigenesis [33]. CDK6 is a member of the cyclin-dependent kinase cdc2 family, whose amino terminal has threonine/serine kinase activity and is an important intracellular cell cycle regulator [34]. Previous studies have elucidated that CDK6 could phosphorylate Rb protein, induces the expression of E2F1, thus inducing the transformation from G1 to S phase, promoting cell proliferation and acting as a tumor promotor in human cancer [34]. Indeed, CDK6 has been found to be abnormally expressed in various types of malignant tumors such as bladder cancer [35] and pancreatic cancer [36], and be a mediator in the regulation of them. For example, CDK6 was recognized as a target of lncRNA HNF1A-AS1/miR-149-5p axis in the modulation of cell proliferation, cell cycle, invasion, and migration of non-small cell lung cancer cells [37]. Jia et al. showed that miR-1296-5p exerted its tumor suppression function through targeting EGFR and CDK6 in gastric cancer [38]. The study of Xue et al. also suggested CDK6 as the downstream target of hsa_circ_0081143/miR-646 axis in gastric cancer, which was capable of regulating development and cisplatin-resistance of gastric cancer [39]. In this study, among the DEGs screened by a gene expression array, CDK6 was identified as a potential target of the UAP1L1 induced regulation of gastric cancer. The upregulation of CDK6 in gastric cancer and its significant association with patient survival were illustrated by experiments or data mining, which was also in agreement with previous reports. More importantly, we found that knockdown of CDK6 could not only directly restrain the development of gastric cancer, but also alleviate even reverse the UAP1L1 overexpression induced promotion of gastric cancer.

In conclusion, this study revealed the abnormal overexpression of UAP1L1 and CDK6 in gastric cancer and the role of UAP1L1/CDK6 axis in gastric cancer progression. UAP1L1 was identified as a potential tumor promotor for gastric cancer which possesses the potential to be used as a therapeutic target in the development of more effective treatment for gastric cancer.

## MATERIALS AND METHODS

### Cell culture

Human gastric adenocarcinoma cell lines MGC-803 and AGS were purchased from BeNa Technology (Hangzhou, Zhejiang, China). MGC-803 cells were cultured in Dulbecco’s Modified Eagle’s Medium (DMEH-214.5g/Liter Glucose) with 10% fetal bovine serum (FBS). AGS were maintained in 90% F-12K with 10% FBS additive. BGC-823 and SGC-7901 were obtained from the American Type Culture Collection (Manassas, VA, USA), and cultured in 90% RPMI-1640 with 10% FBS. All cells were maintained at 37°C with 5% CO_2_.

### Immunohistochemistry analysis

Human gastric cancer tissues and adjacent normal tissues were obtained during the surgery from gastric cancer patients. Totally 40 pairs were collected and age of these patients were ranged from 23 to 40 year-old. Patients were informed before the surgery and related clinical information was collected. The study design was approved by Medical Ethics Committee of the Xiangya Hospital of Central South University. Fresh gastric cancer tissues and normal tissues were fixed using 4% paraformaldehyde (Sigma, St Louis, MO, USA) and embedded in paraffin. For immunohistochemistry analysis, the tissue specimens were dewaxed in xylene and dehydrated in ascending series of ethanol. EDTA buffer were added for antigen repair in the boiling water for 30 min. All tissue specimens’ endogenous peroxidase was blocked with 3% H_2_O_2_ for 5 min. After blocked with rabbit serum, the sections were incubated with anti-UAP1L1 at 4°C overnight and then incubated with the secondary antibody for 2 h at room temperature. Finally, the tissue specimens were stained with diaminobenzidine and exanimated with microscopic at the magnification of x200 and x400. Specimens were classified into four categories: negative (0), positive (1-4), ++ positive (5-8), or +++ positive (9-12), based on the sum of the staining intensity (varied from weak to strong) and staining extent scores, which graded as 0 (0%), 1 (1-25%), 2 (26-50%), 3 (51-75%), or 4 (76-100%). Antibodies used were showed in Supplementary Table 1.

### Plasmid construction, lentivirus infection and transfection

Using UAP1L1 and CDK6 gene as template, overexpression and knockdown sequences were designed by Shanghai Bioscienceres Co., Ltd. (Shanghai, China). Target sequences for UAP1L1 (5’-GCCCTTCTTACTGCAAACCAT-3’) and CDK6 (5’-AAGGATATGATGTTTCAGCTT-3’; 5’-TGGCTGCATATTTGCAGAAAT-3’; 5’-GCCCAACCAATTGAGAAGTTT-3’) were inserted into in BR-V-108 vector and transformed into E. coli competent cells (Tiangen, Beijing, China). EndoFree maxi plasmid kit (Tiangen, Beijing, China) was used for plasmid extraction according to the manufacturer’s instruction. The 293T cells were transfected with qualified UAP1L1 or CDK6 plasmids using Lipofectamine 2000 transfection reagent (Thermo Fisher Scientific, Waltham, MA, USA) and target lentivirus were packaged in lentivirus production.

400 μL 1×10^7^ TU/mL lentivirus were transfected into logarithmic growth phase BGC-823 and SGC-7901 cells in a 6-well dish with 2×10^5^ cells per well. Transfected cells were cultured in complete medium for 72 h prior to use in the apoptosis and migration and experiments and cell infection efficiency was evaluated by microscopic fluorescence (Olympus, Tokyo, Japan).

### RNA extraction and RT-qPCR

Total RNA from transfected SGC-7901, AGS, MGC-803 and BGC-823 cells were extracted using TRIzol reagent (Sigma, St. Louis, MO, USA) and the quality of total RNA was evaluated by Nanodrop 2000 spectrophotometer (Thermo Fisher Scientific, Waltham, MA, USA) according to the manufacturer’s instructions. cDNA was synthesized using RNA (2.0 μg) according to the instructions of Vazyme’s Hiscript QRT Supermix for qPCR and quantitative real-time PCR was conducted with SYBR Green Mastermixs Kit (Vazyme, Nangjing, Jiangsu, China) on the platform of Applied Biosystems 7500 Real-Time PCR system. GAPDH was utilized as inner control and the related primers used for the PCR reaction were showed in Supplementary Table 2.

### MTT assay

Transfected cells BGC-823 and SGC-7901 with or without exposure to UAP1L1 in exponential growth phase were trypsinized and seeded into a 96-well plate with 2,000 cells per well. Following incubation at 37°C for 24 h, 48 h, 72 h, 96 h and 120 h, 20 μL MTT solution (5 mg/mL, GenView, El Monte, CA, USA) was added and incubated for 4 h and then 100 μL dimethyl sulphoxide solution was added. The absorbance values at 490 nm were measured by a microplate reader (Tecan, Männedorf, Zürich, Switzerland) and cell viability was detected by MTT assay according the manufacturer’s protocol.

### Western blotting (WB) assay and Co-immunoprecipitation (co-IP)

Transfected cells were lysed in ice-cold RIPA buffer (Millipore, Temecula, CA, USA) and proteins were homogenized. Total protein concentration was detected by bicinchoninic acid (BCA) Protein Assay Kit (HyClone-Pierce, Logan, UT, USA). Equal amounts of proteins were separated by 10% SDS-polyacrylamide gel electrophoresis (PAGE) (Invitrogen, Carlsbad, CA, USA) and were transferred onto preparation and modification of polyvinylidene fluoride (PVDF) membranes. All membranes were incubated with primary antibodies at 4°C overnight and continuingly incubated with the secondary antibody for 2 h at room temperature. The blots were visualized by enhanced chemiluminescence (ECL) (Amersham, Chicago, IL, USA).

For co-IP, cell lysate was prepared as WB assay, and 1.0-1.2 mg proteins were incubated with normal rabbit IgG (as control) for 2 h, and followed by 2 h of incubation with 20 μL protein A/G-beads. The cleared protein antibody beads complex was incubated at 100°C for 10 min. Then the proteins in the immunocomplex were separated by 10% SDS-PAGE as WB assay, and used for immunoblotting to identify interacting proteins.

### Human apoptosis antibody array

For detection of related genes in human apoptosis signaling pathway, Human Apoptosis Antibody Array (R&D Systems, Minneapolis, MN, USA) was applied following the manufacturer’s instructions. Briefly, total proteins from the transfected SGC-7901 cells were extracted and protein concentrations were measured. Each array antibody membrane was blocked, then incubated with protein samples (0.5 mg/mL) overnight at 4°C and continuing incubated with secondary antibody for 1 h. The spots were visualized by enhanced chemiluminescence (ECL) and the signal densities were analyzed with ImageJ software (National Institute of Health, Bethesda, MD, USA). Antibodies used were detailed in Supplementary Table 1.

### Celigo cell counting assay

Transfected SGC-7901 cells were collected and seeded into 96-well plates (2,000 cells/well) in RPMI-1640 medium containing 10% FBS at 37°C with 5% CO_2_ for 120 h. Cell counting was accomplished by Celigo image cytometer (Nexcelom Bioscience, Lawrence, MA, USA) at day 1, 2, 3, 4 and 5 and the cell proliferation was evaluated.

### Flow cytometry for apoptosis and cell cycle

The flow cytometric methods of identifying apoptotic cells-Annexin V-FITC Apoptosis kit (eBioscience, San Diego, CA, USA) were applied here. Lentivirus transfected cells were inoculated in 6-well plates with 2,000 cells per well in triplicate and further cultured for 5 days. Cells were collected, trypsinized and washed with 4°C ice-cold D-Hanks. After centrifugation (1300 × g) for 3 min, cells were resuspended with binding buffer, then 10 μL Annexin V-APC was added for staining without light. Apoptosis analysis was measured using flow cytometry Guava easyCyte HT (Millipore, Schwalbach, Germany).

For cell cycle detection, cells were stained with 1.5 mL cell staining solution (40 × Propidium Iodide (2 mg/mL) (Sigma, St Louis, MO, USA): 100 × RNase (10 mg/mL): 1 × PBS =25: 10: 1000). Cell cycle distribution was detected by flow cytometry (200~350 Cell/s) and observed by IX73 micropublisher (Olympus, Tokyo, Japan).

### Wound healing assay

Lentivirus transfected BGC-823 and SGC-7901 cells were seeded at 5 × 10^4^ cells per well onto a 96-well dish. After synchronization, scratch was made by a 96-wounding replicator (VP scientific, San Diego, CA, USA) across the cell layer, the floating cells were washed away. RPMI-1640 medium with 0.5% FBS was added for culturing and photographs were taken by a fluorescence microscope at 0 h, 8 h and 24 h after scratching. Cell migration rate of each group was calculated.

### Colony formation assay

Transfected BGC-823 and SGC-7901 cells were seeded into 6-well plates at 500 cells per well in triplicate. The RPMI-1640 culture medium with 10% FBS was exchanged every 3 days. The cell clones were photographed under a fluorescence microscope. Then all clones were fixed by 4% paraformaldehyde and stained by Giemsa. The cell clones were photographed with a digital camera. Colony forming rate = (colony number / inoculated cell number) × 100%.

### Transwell assay

Migration potential of transfected BGC-823 and SGC-7901 cells was quantified by Transwell assay using Corning Transwell Kit (Corning, NT, USA). First, exponentially growing cells were seeded in the upper chamber with 100 μL medium without FBS in a 24-well plate (5×10^4^ cells/well). 600 μL medium supplemented with 30% FBS was added in the lower chamber. Cells were incubated for 24 h at 37°C with 5% CO_2_ and non-metastatic cells were removed. After cells were fixed by 4% paraformaldehyde, 400 μL Giemsa was added for staining and the cell migration ability was quantified by fluorimetry micropublisher 3.3RTV (Olympus, Tokyo, Japan) and Biotek Elx800 Microplate Reader (Winooski, VT, USA).

### PrimeView human gene expression array

Gene expression in transfected SGC-7901 cells was detection by RNA screening analysis in Shanghai Bioscienceres, Co., Ltd. (Shanghai, China). Briefly, total RNA was extracted by the RNeasy kit (Sigma, St. Louis, MO, USA). Quality and integrity of total RNA was determined by Nanodrop 2000 (Thremo Fisher Scientific, Waltham, MA, USA) and Agilent 2100 and Agilent RNA 6000 Nano Kit (Agilent, Santa Clara, CA, USA). RNA sequencing was performed with Affymetrix human GeneChip PrimeView according to the manufacturer’s instruction and the outcomes were scanned by Affymetrix Scanner 3000 (Affymetrix, Santa Clara, CA, USA). Raw data statistical significance assessment was accomplished using a Welch t-test with Benjamini-Hochberg FDR (|Fold Change| ≥ 2.0 and FDR < 0.05 as significant). Significant difference analysis and functional analysis based on Ingenuity Pathway Analysis (IPA) (Qiagen, Hilden, Germany) was executed, and |Z - score| > 2 is considered meaningful.

### *In vivo* tumorigenicity assay

20 female 4-week-old BALB/c nude mice were purchased from Shanghai Lingchang Experimental Animals Co., Ltd (Shanghai, China). Mice were randomly divided into shUAP1L1 group and shCtrl group. 4 × 10^7^ transfected SGC-7901 cells were subcutaneous injected into each mouse on the right axillary for *in vivo* tumorigenicity. One week later, we started collected the data of mice’ weight, L and W of tumors (L represent longest dimension and W means dimension perpendicular to length, and tumor volume was calculated as π/6×L×W^2^) and data collecting frequency was 2 times per week. Bioluminescence imaging was applied by IVIS Spectrum Imaging System (Perkin Elmer, Waltham, MA, USA). Three weeks post cell injection, all mice were sacrificed and the tumor tissues were removed and pictures were photographed. This animal study was reviewed and approved by Medical Ethics Committee of the Xiangya Hospital of Central South University.

### Statistical analysis

All cell experiments were repeated three times and outcomes were expressed as the mean ± SD and Student’s T-Test or one-way ANOVA was used to analyze the statistical significance using SPSS 19.0 (IBM, SPSS, Chicago, IL, USA) and GraphPad Prism 6.01 (Graphpad Software, La Jolla, CA, USA). A *P*-Value of < 0.05 was considered statistically significant. Categorical variables were compared using χ^2^. Relationship between UAP1L1 expression and clinical tumor characteristics in gastric were analyzed by Mann-Whitney U analysis. Expression patterns in gastric cancer tissues and normal tissues revealed in immunohistochemistry analysis accomplished with Spearman Rank correlation analysis. The relative quantitative analysis in gene expression data were analyzed by the 2^−ΔΔCt^ method.

## Supplementary Material

Supplementary Figures

Supplementary Tables
