# Supplementary material for: Identification of UAP1L1 as tumor promotor in gastric cancer through regulation of CDK6
Source: Aging (Albany NY). 2020 Apr 20;12(8):6904–27. doi: 10.18632/aging.103050 (PMC7202507; doi:10.18632/aging.103050)
Supplement: Supplementary Figures [file aging-12-103050-s002..pdf]

SUPPLEMENTARY FIGURES

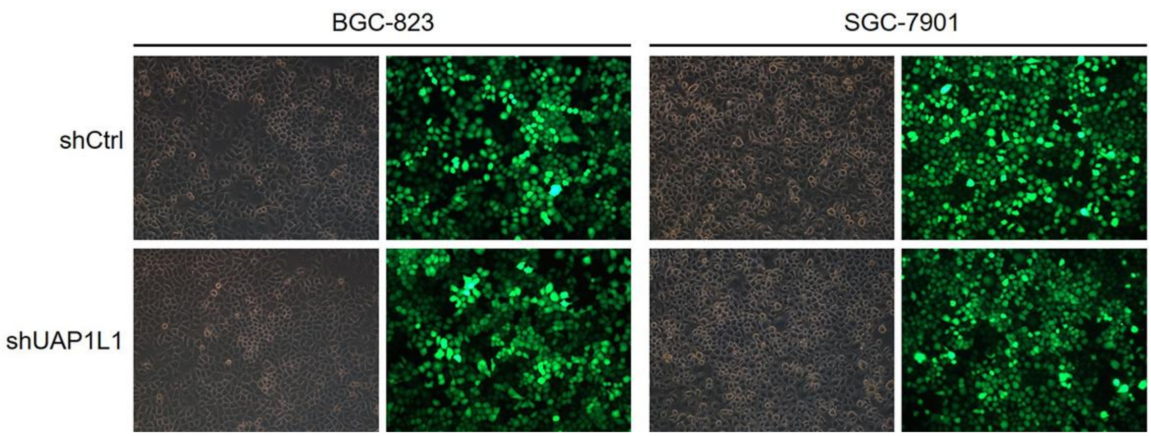

Supplementary Figure 1. The transfection efficiencies of shUAP1L1 and shCtrl in BGC-823 and SGC-7901 cells were evaluated through observing the fluorescence of GFP on lentivirus vector.

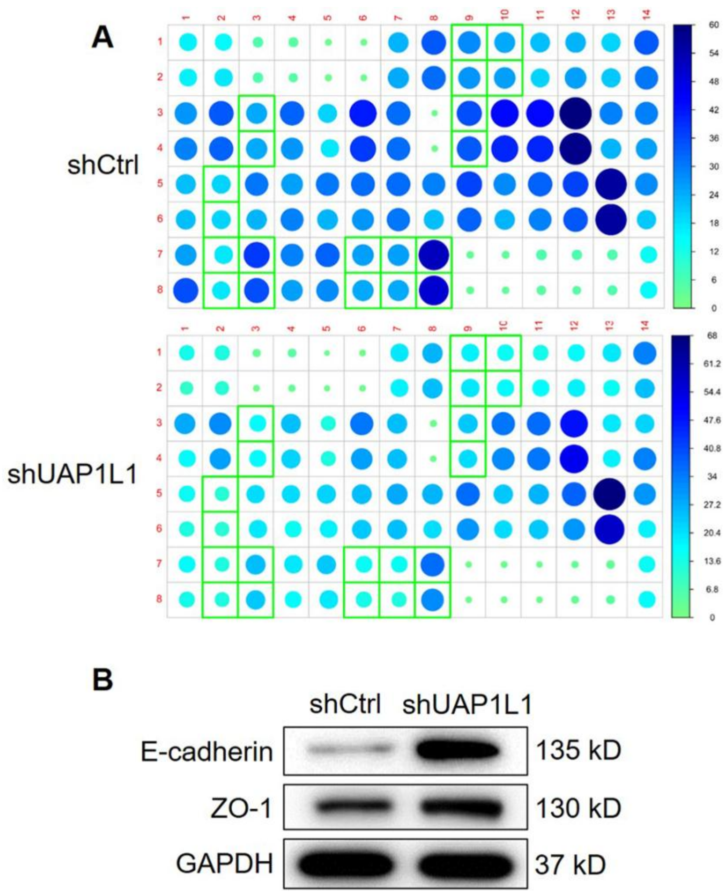

Supplementary Figure 2. (**A**) Human Apoptosis Antibody Array was performed to detect and compare the expression of apoptosis-related proteins in SGC-7901 cells with or without UAP1L1 knockdown. (**B**) The expression of E-cadherin and ZO-1 in BGC-823 cells with or without UAP1L1 knockdown was detected by western blotting.

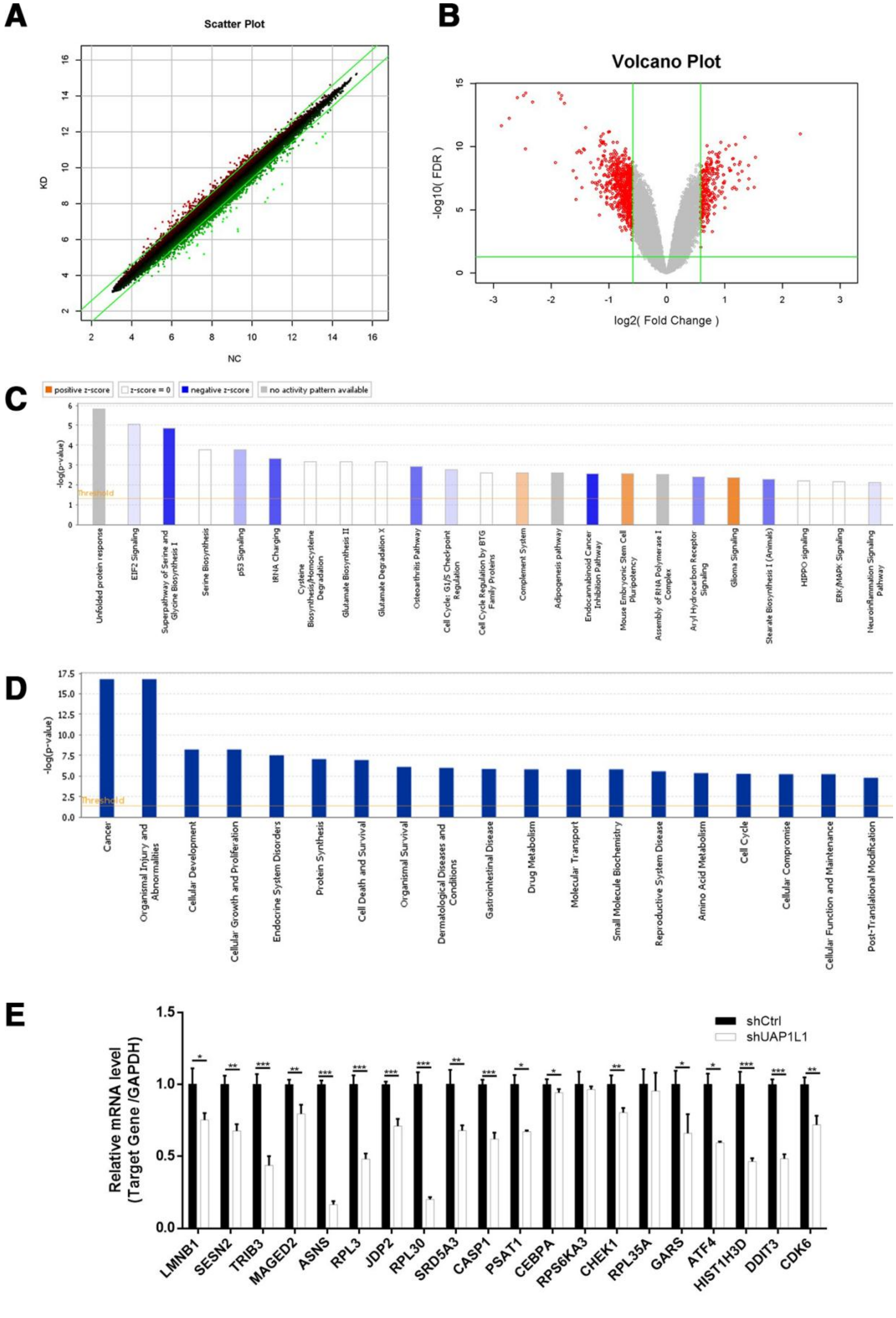

Supplementary Figure 3. (**A**) The scatter plot of gene expression profiling in SGC-7901 cells with or without UAP1L1 knockdown. Red dots represented significantly upregulated DEGs. Green dots represented significantly downregulated DEGs. (**B**) The volcano plot of gene expression profiling in SGC-7901 cells with or without UAP1L1 knockdown. Red dots represented the DEGs. (**C**) The enrichment of the DEGs in canonical signaling pathways was analyzed by IPA. (**D**) The enrichment of the DEGs in IPA disease and function was analyzed by IPA. (**E**) The mRNA expression of 20 selected DEGs was detected by qPCR in BGC-823 cells with or without UAP1L1 knockdown. Data was shown as mean ± SD. **P* < 0.05, ***P* < 0.01, ****P* < 0.001.

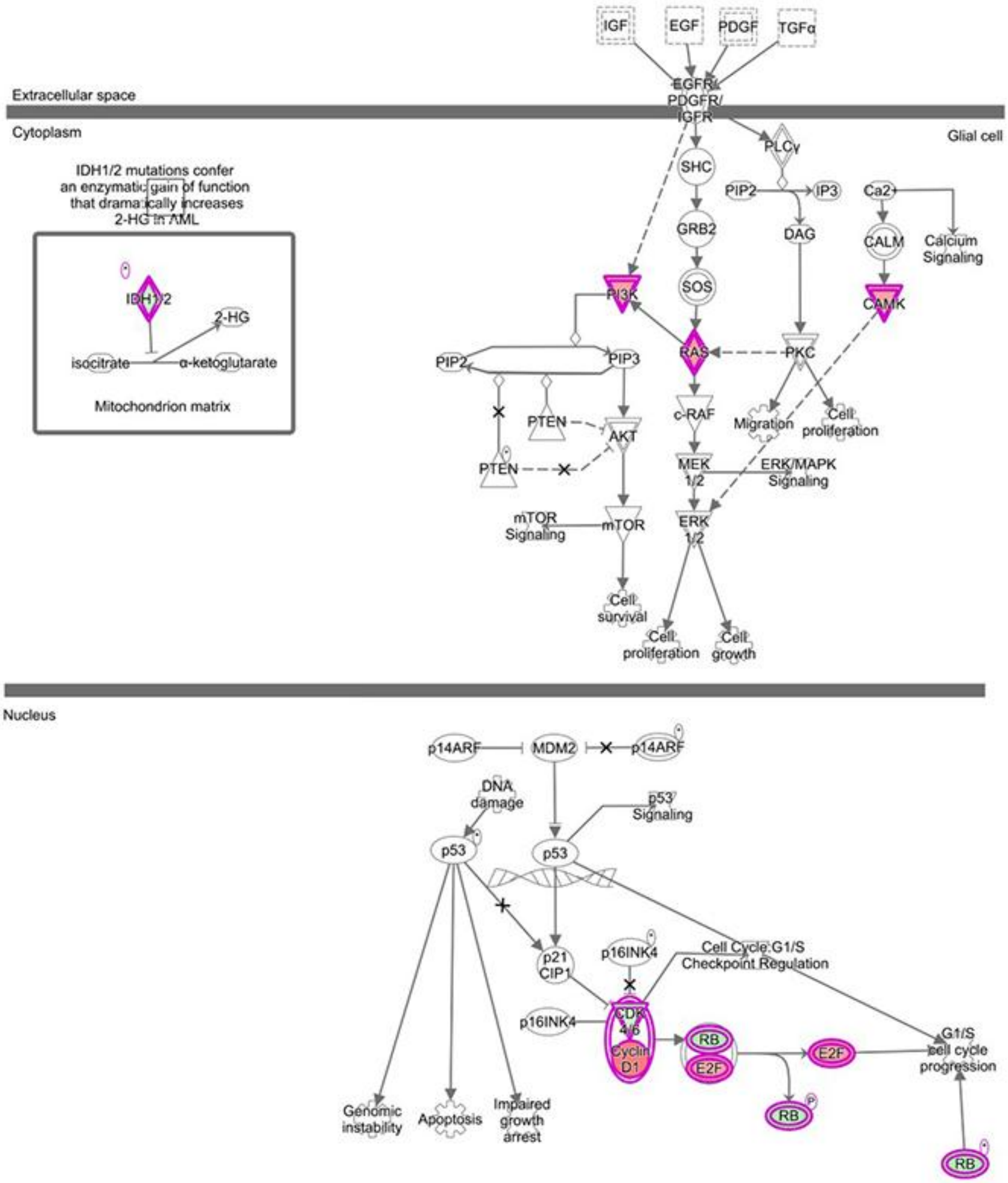

Supplementary Figure 4. The schematic diagram of Glioma signaling.

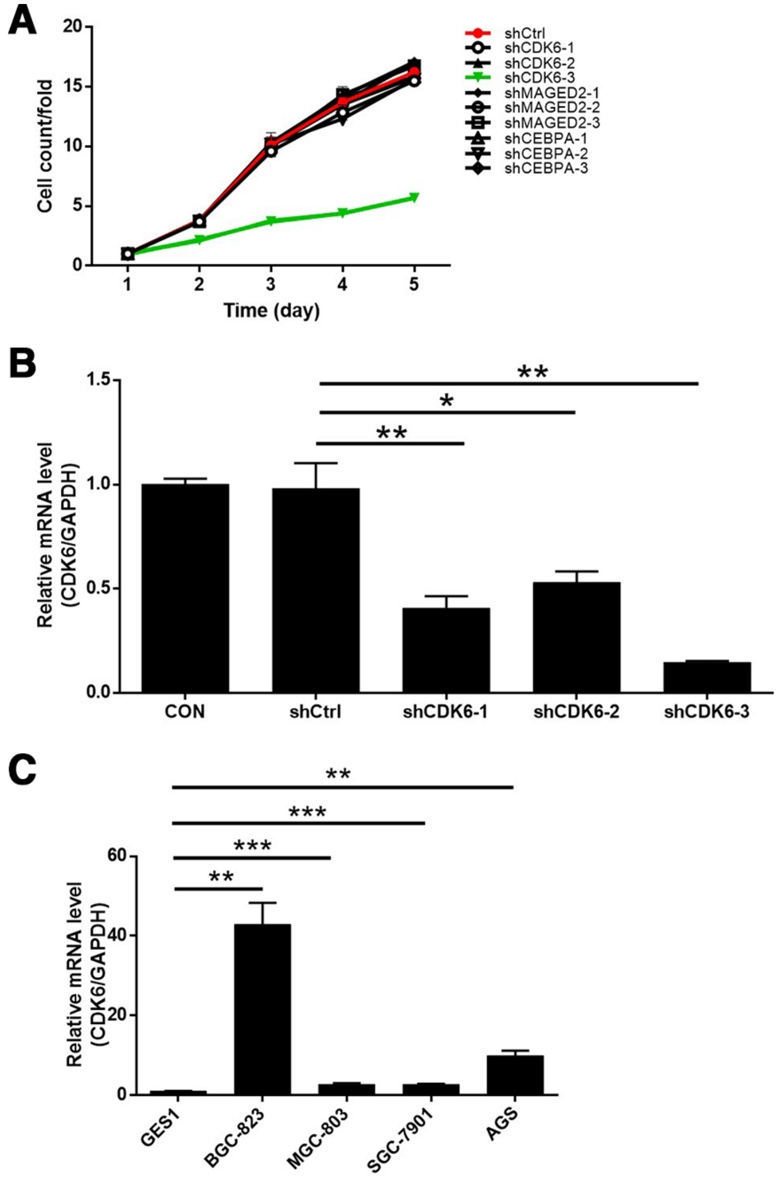

Supplementary Figure 5. (**A**) Celigo cell counting assay was performed to evaluate the inhibitory ability of lentivirus expressing different shRNAs targeting CDK6, MAGED and CEBPA on SGC-7901 cell proliferation. (**B**) The knockdown efficiencies of 3 shRNAs targeting CDK6 were assessed by qPCR. (**C**) The mRNA expression of UAP1L1 in GES-1, BGC-823, SGC-7901, AGS and MGC-803 cell lines was detected by qPCR. Data was shown as mean ± SD. *$P < 0.05$, **$P < 0.01$, ***$P < 0.001$.

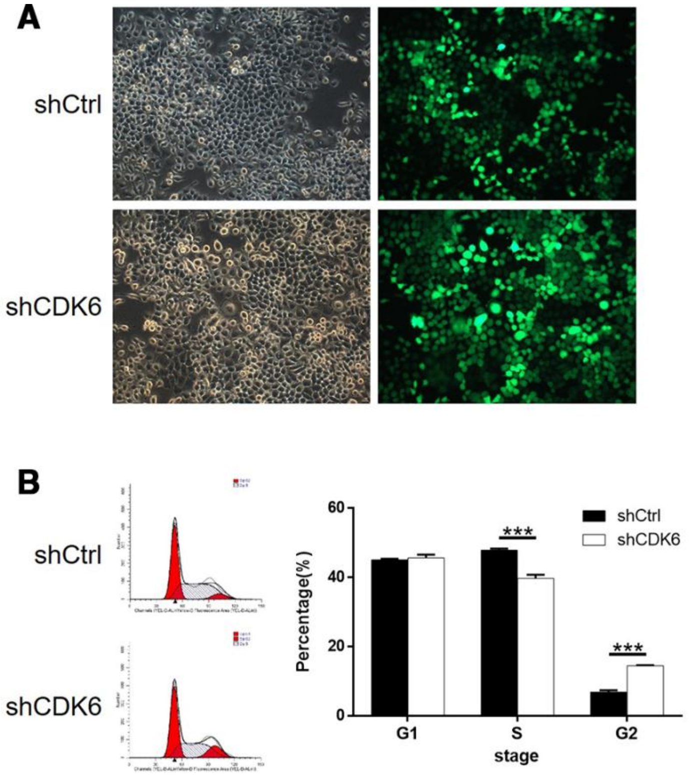

Supplementary Figure 6. (**A**) The transfection efficiencies of shCtrl and shCDK6 were evaluated through observing the fluorescence of GFP on lentivirus vector. (**B**) The effects of CDK6 knockdown on cell cycle distribution of SGC-7901 were detected by flow cytometry. Data was shown as mean ± SD. ****P* < 0.001.

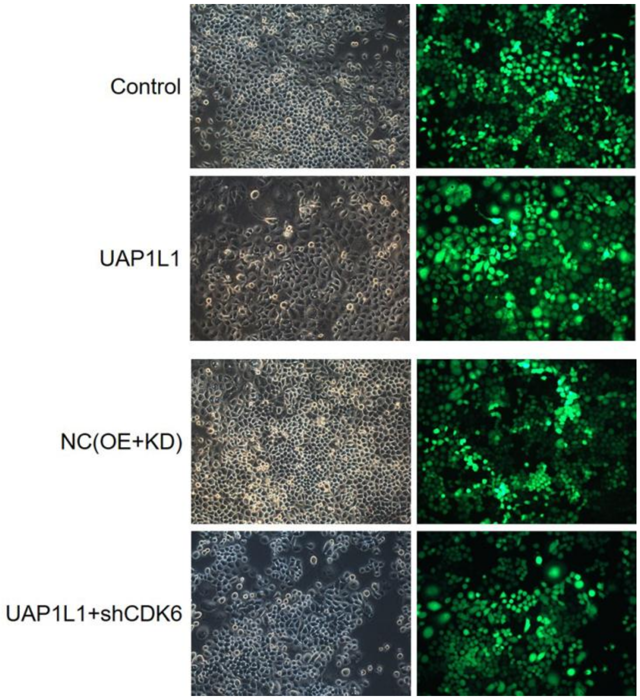

Supplementary Figure 7. The transfection efficiencies of Control plasmid, UAP1L1 overexpression plasmid, NC(OE+KD) and UAP1L1+shCDK6 in SGC-7901 cells were evaluated through observing the fluorescence of GFP on lentivirus vector.

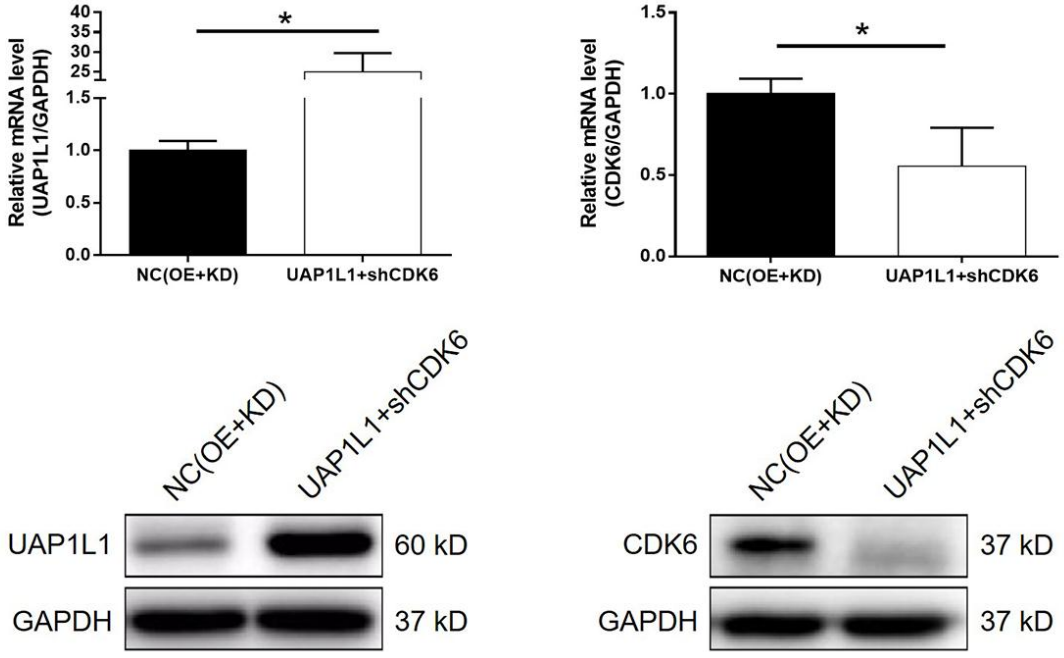

Supplementary Figure 8. The mRNA and protein levels of UAP1L1 and CDK6 in SGC-7901 cells transfected with NC(OE+KD) or UAP1L1+shCDK6 were detected by qPCR and western blotting, respectively. Data was shown as mean ± SD. **P* < 0.05.
