# Supplementary material for: Identification of UAP1L1 as tumor promotor in gastric cancer through regulation of CDK6
Source: Aging (Albany NY). 2020 Apr 20;12(8):6904–27. doi: 10.18632/aging.103050 (PMC7202507; doi:10.18632/aging.103050)
Supplement: Supplementary Tables [file aging-12-103050-s001..pdf]

SUPPLEMENTARY TABLES

Supplementary Table 1. Antibodies used in western blotting and IHC.

Primary antibodies	Dilution in WB	Source species	Company	Catalog No.
UAP1L1	1:1000	Rabbit	abcam	ab174855
GAPDH	1:3000	Rabbit	Bioworld	AP0063
N-cadherin	1:1000	Rabbit	abcam	ab18203
E-cadherin	1:1000	Rabbit	CST	3195s
ZO-1	1:1000	Rabbit	abcam	ab214228
Vimentin	1:1000	Rabbit	abcam	ab92547
Snail	1:1000	Rabbit	CST	3879S
CDK6	1:1000	Rabbit	abcam	ab151247
MAGED2	1:1000	Rabbit	abcam	ab236592
RPL35A	1:1000	Rabbit	biorbyt	orb513214
TRIB3	1:1000	Rabbit	abcam	ab73547
CEBPA	1:1000	Rabbit	abcam	ab40764
DYKDDDDK Tag*	1:50/1:1000	Rabbit	CST	14793
* DYKDDDDK Tag binds to same epitope as Sigma's Anti-FLAG® M2 Antibody				
**Primary antibodies**	**Dilution in IHC**	**Source species**	**Company**	**Catalog No.**
UAP1L1	1:200	Rabbit	abcam	ab174855
CDK6	1:100	Rabbit	abcam	ab151247
Secondary antibody	Dilution		Company	Catalog No.
HRP Goat Anti-Rabbit IgG (WB)	1:3000		Beyotime	A0208
HRP Goat Anti-Rabbit IgG (IHC)	1:200		Abcam	Ab111909

Supplementary Table 2. Primers used in qPCR.

Gene	Forward primer sequence (5'-3')	Reverse primer sequence (5'-3')
GAPDH	TGACTTCAACAGCGACACCCA	CACCCTGTTGCTGTAGCCAAA
UAP1L1	GGAGCGGAAAGACAAAGTTGC	CACAGAAGCCGATGAAGACAGG
LMNB1	CCCAGTTGGAAGCCTCCTTA	GCGAAACTCCAAGTCCTCAG
SESN2	ATAGCCTCACCTACAATACCATCG	CACCTCCCCATAATCATAGTCATC
TRIB3	AGCGGTTGGAGTTGGATGA	TTGCACGATCTGGAGCAGTAG
MAGED2	TCCAGTCATCCCAAGAGCCT	AATCTTCGTCTGGTCTTTAGCC
ASNS	GCACGCCCTCTATGACAATG	CTGATAAAAGGCAGCCAATCCT
RPL3	AGAGGCTTGAGCAGCAGGTA	ACGACTGGTGACCCCTTTGT
JDP2	CACTCCTCCTGCTATGATGCCT	TGCGGATGTCAGCGTATTTC
RPL30	GGTACAAGCAGACTCTGAAGATGA	TGATGGACACCAGTTTTAGCC
SRD5A3	CCAATGGATGGCAGGAATG	TGGGCAGATGACCAGATGAAC
CASP1	ATGCCTGTTCCTGTGATGTGG	AAGTCACTCTTTCAGTGGTGGG
PSAT1	GATTGTCCGTGATGACCTGC	AGATGCTGAAACATGGAGGC
CEBPA	CTTGGTGCGTCTAAGATGAGG	ATTGGAGCGGTGAGTTTGC
RPS6KA3	ATTGGCACGAATAGGTAGCG	GCATCTTTGACACCAGGTCC
CHEK1	TTGGCTTGGCAACAGTATTTCG	CCAGCGAGCATTGCAGTAAGT
RPL35A	GAAGGTGTTTACGCCCGAGAT	CGAGTTACTTTTCCCCAGATGAC
GARS	CGCATCTACCTCTACCTCACGA	TCCCAACAGTCACAGGCATAA
ATF4	CCCTTCACCTTCTTACAACCTC	TTCACTGCCCAGCTCTAAACTA
HIST1H3D	CAACGACGAGGAGCTAAACAA	GCCATTGCGAACTTCTAAACC
DDIT3	GAGCTGGAAGCCTGGTATGA	AGAAGCAGGGTCAAGAGTGGT
CDK6	TCCCAGGCAGGCTTTTCAT	GGGCACTGTAGGCAGATATTCTT

Supplementary Table 3. Relationship between UAP1L1 expression and tumor characteristics in patients with gastric cancer analyzed by Spearman rank correlation analysis.

Tumor characteristics	index	
T Infiltrate	Pearson correlation	0.439
	Significance (two tailed)	0.005**
	n	40
